# Periodic mild heat stimuli diminish extracellular matrix synthesis in pellet cultured human chondrocytes

**DOI:** 10.1186/s13104-019-4058-x

**Published:** 2019-01-14

**Authors:** Akira Ito, Tomoki Aoyama, Hirotaka Iijima, Kohei Nishitani, Junichi Tajino, Hiroshi Kuroki

**Affiliations:** 1 Department of Motor Function Analysis, Human Health Sciences, Graduate School of Medicine, Kyoto University, 53 Kawahara-cho, Sakyo-ku, Shogoin, Kyoto 606-8507 Japan; 2 Department of Development and Rehabilitation of Motor Function, Human Health Sciences, Graduate School of Medicine, Kyoto University, Kyoto, 606-8507 Japan; 3 Department of System Design Engineering, Faculty of Science and Technology, Keio University, Yokohama, 223-8522 Japan; 4 Department of Orthopaedic Surgery, Graduate School of Medicine, Kyoto University, Kyoto, 606-8501 Japan

**Keywords:** Heat stimuli, Chondrocytes, Extracellular matrix, Collagen, Pellet culture, Temperature

## Abstract

**Objective:**

This study aimed to clarify the effects of periodic mild heat stimuli on extracellular matrix (ECM) synthesis of adult human chondrocytes in 3-dimensional pellet culture.

**Results:**

Human articular chondrocytes were subjected to pellet culture at 37 °C for 3 days. Thereafter, the pellets were divided into three groups: 32 °C group which was cultured at 32 °C without heat stimuli, 32 °C + Heat group which was cultured at 32 °C and applied periodic heat stimuli, 37 °C group which was cultured at 37 °C. Heat stimuli were given by transferring the pellets into a CO_2_ incubator set at 41 °C for 20 min/day, 6 times/week. ECM synthesis ability was evaluated by analyzing the mRNA expressions. Additionally, the collagen and proteoglycan content in the pellet was quantified. DNA content was also measured for estimating the cell amount. We found that there were no significant differences in the mRNA expression of COL2A1, COL1A1, and ACAN between the 32 °C group and 32 °C + Heat group. However, the collagen content per cell and DNA content were significantly lower in the 32 °C + Heat group compared to other groups. Our results indicate that periodic mild heat stimuli may diminish ECM synthesis due to inhibition of collagen production and loss of cells.

**Electronic supplementary material:**

The online version of this article (10.1186/s13104-019-4058-x) contains supplementary material, which is available to authorized users.

## Introduction

Articular cartilage (AC) defects caused by factors such as trauma lead to pain, malfunction, and eventual onset osteoarthritis. Unfortunately, AC cannot regenerate spontaneously; therefore, several treatments have been developed for AC regeneration, including cell-based therapy [1, 2]. Autologous chondrocyte implantation (ACI) is one of the promising cell-based therapies for regeneration of AC defects [3]. However, ACI still has several challenges. Chondrocytes cultured in vitro undergo dedifferentiation, causing fibro-cartilage like remodeling [4]. Furthermore, the extracellular matrix (ECM) around the implanted chondrocytes needs enough maturation time for constructing firm structures to resist load bearing and takes a long time (8–12 weeks) to obtain full weight bearing capacity [5]. In order to enhance ECM synthesis and maturation, microenvironmental factors such as the presence of growth factors, scaffolds, oxygen tension, and mechanical stimuli have been intensively investigated for several decades [6–9]. In addition to these factors, we recently proposed the importance of temperature for ECM synthesis by chondrocytes [10, 11]. Because the temperature within the human knee joint is influenced by environmental temperature, the mean temperature of the human knee joint is approximately 32 °C, which is 4–5 °C lower than the inner body temperature [12, 13]. We reported that culture temperatures affected ECM synthesis of chondrocytes in a three-dimensional (3-D) pellet culture system using adult human chondrocytes [11], and found that a culture temperature of 37 °C was superior to that of 32 °C for ECM synthesis. This indicates that temperature modulation could be used as a treatment to enhance ECM synthesis and maturation for ACI. However, to the best of our knowledge, there have been no studies investigating the effects of periodic heat stimuli on the ECM synthesis of AC in human chondrocytes. Here we hypothesized that periodic mild heat stimuli enhance ECM synthesis in adult human chondrocytes. This study aimed to clarify the effects of periodic mild heat stimuli on ECM synthesis of adult human chondrocytes in a 3-D pellet culture system.

## Main text

### Materials and methods

#### Chondrocyte isolation

Human chondrocytes were isolated from the articular cartilage of a femoral head extracted during a bipolar hip arthroplasty on an 89-year-old woman using a previously described method [14]. The Ethics Committee of the Faculty of Medicine, Kyoto University approved the procedure (approval no. 944), and informed consent was obtained from the donor. The isolated cells were cultured until passage two.

#### Pellet culture

To provide a 3-D environment and to mimic in vivo ECM synthesis, a pellet culture system was used in this study. The expanded chondrocytes were resuspended in chondrogenic medium (chondrogenic basal medium plus ITS + supplement, ascorbate, dexamethasone, l-glutamine, sodium pyruvate, proline, and GA-1000; Lonza; Walkersville, MD) supplemented with 10 ng/mL of recombinant human transforming growth factor-beta 3 (R&D Systems; Minneapolis, MN). Aliquots of 2.5 × 10^5^ cells in chondrogenic medium were centrifuged at 250×*g* for 5 min in 15-mL polypropylene conical tubes, and then pre-cultured at 37 °C in a CO_2_ incubator for 3 days. Throughout the study, “*n*” indicates the technical replicates of the pellet cultures.

#### Periodic heat stimuli

**Fig. 1:**
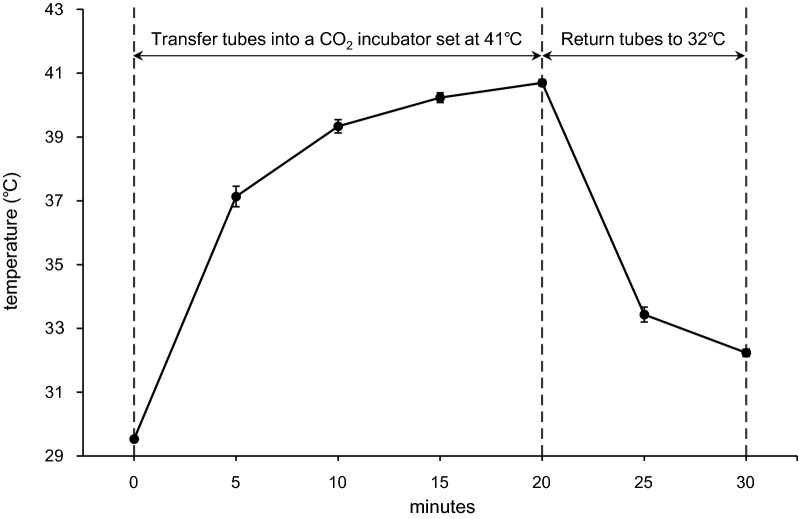
Temperature changes. The temperature of the medium in a 15-mL polypropylene conical tube was measured for 30 min using a digital thermometer. The tube with 500 µL of medium was transferred into a CO_2_ incubator set at 41 °C for 20 min for heat stimulation, and then placed back into a CO_2_ incubator set at 32 °C for another 10 min. The temperature within the medium was over 40 °C at 15 min, and immediately dropped in 10 min after the heat stimulus. This experiment was repeated 3 times, and values represent the means and standard deviations

The pre-cultured pellets were divided into three groups: the 32 °C group which was cultured at 32 °C without heat stimuli, 32 °C + Heat group which was cultured at 32 °C and applied periodic heat stimuli, and the 37 °C group which was cultured at 37 °C as a control. We utilized 41 °C as a heat stimulus because it is frequently used as an effective and safe thermal therapy for patients and animal experiments. Heat stimuli were given by transferring the pellets into a separate CO_2_ incubator set at 41 °C for 20 min/day, 6 times/week. The temperature transition of culture medium in the conical tubes was monitored using a digital thermometer (BAT-7001H Thermometer, Physitemp Instruments Inc.; Clifton, NJ) and is shown in Fig. 1.

#### mRNA expression related to extracellular matrix in articular cartilage

In order to assess the immediate effects of heat stimulation on ECM synthesis, ECM-related mRNA expression of AC was evaluated. The pre-cultured pellets at 37 °C were transferred into a separate CO_2_ incubator set at 32 °C for 24 h or kept in a CO_2_ incubator set at 37 °C as controls. Thereafter, heat stimulus was applied by transferring the pellets into a CO_2_ incubator set at 41 °C for 20 min. After heat stimulation, the pellets were placed back into the CO_2_ incubator set at 32 °C. Total mRNA was extracted from the heat stimulated and control pellets before and at 1, 6, and 24 h after heat stimulation (n = 3 pellets/group condition). The mRNA expression of heat shock 70 kD protein 1A (HSPA1A), type II collagen A1 (COL2A1), type I collagen A1 (COL1A1), and aggrecan (ACAN) was assessed by quantitative real-time reverse-transcription polymerase chain reaction (qRT-PCR) as described previously [15]. Furthermore, the accumulated effects of heat stimuli on mRNA expression were assessed. The pellets from the three groups were harvested and total mRNA was extracted on day 7 (n = 3 pellets/group). The mRNA expressions were assessed by qRT-PCR. Ribosomal protein L13a (RPL13a) and tyrosine 3-monooxygenase/tryptophan 5-monooxygenase activation protein (YWHAZ) were used as reference genes, which are proven to be stable under different thermal conditions [15]. The gene-specific primers are listed in Additional file 1.

#### Collagen and proteoglycan accumulation

Collagen and proteoglycan accumulation in pellets obtained on day 21 was evaluated histologically and biochemically. The pellets were subjected to macrophotography and wet weight measurement (*n* = 12 pellets/group).The pellets were then used for picrosirius red staining to visualize collagen, and for safranin-O staining to assess proteoglycan deposition (*n* = 6 pellets/group). For quantitative comparison, collagen and proteoglycan content were measured using the hydroxyproline assay [16] for collagen content and the 1,9-dimethylmethylene blue (DMMB) colorimetric assay for proteoglycan content (*n* = 6 pellets/group) [17]. In order to estimate the cell amount in the pellet, DNA content (n = 6 pellets/group) was measured by the Quant-iT™ PicoGreen® assay (Invitrogen Ltd., Paisley, UK) following the manufacturer’s instructions. The values of collagen and proteoglycan were normalized to DNA content in order to describe the amount of these molecules per cell.

#### Statistical analysis

JMP 11 software (SAS Institute, Cary, NC) was used for the statistical analyzes. Statistical significance was determined by one-way analysis of variance following a post-hoc multiple comparison Tukey–Kramer test. The observed differences were considered to be significant if P < 0.05.

### Results

#### Effects of heat stimulus on mRNA expression related to ECM synthesis

**Fig. 2:**
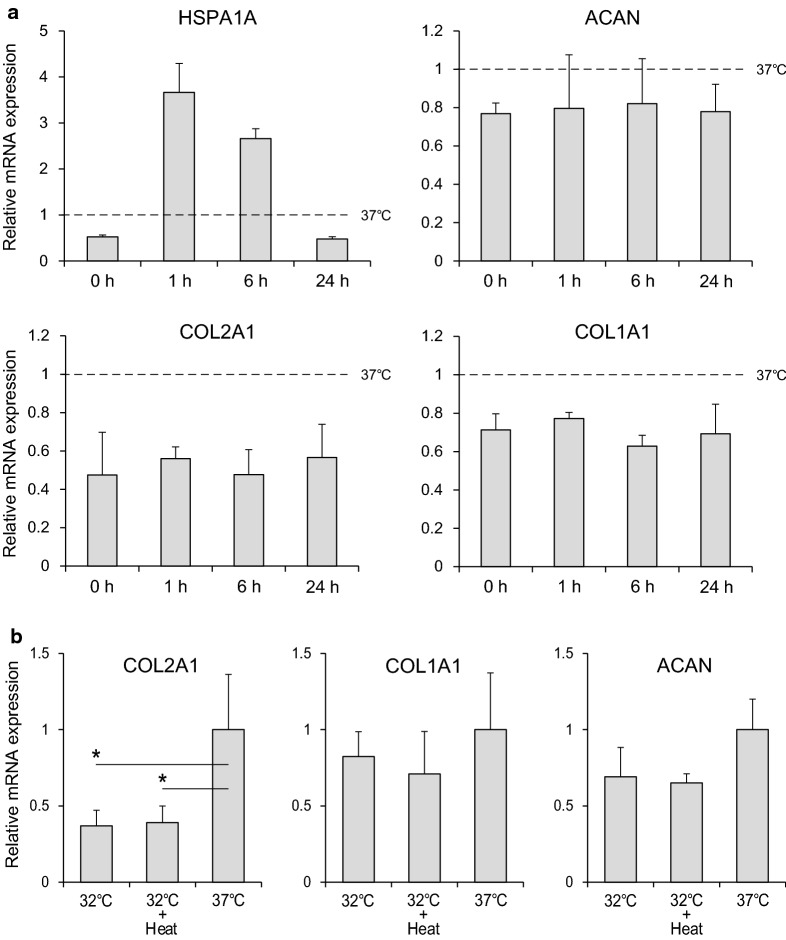
Immediate and accumulative effects of heat stimulus on mRNA expression. **a** Relative mRNA expression of HSPA1A, ACAN, COL2A1, and COL1A1 at 0, 1, 6, 24 h after heat stimulation. **b** Relative mRNA expression of COL2A1, COL1A1, and ACAN on day 7 is shown. Values represent the means and standard deviations. Asterisk indicates P < 0.05

The expression of HSP1A was upregulated in 1 h after heat stimulation, thus confirming the response to heat stimulus (Fig. 2a). However, the expression of COL2A1, COL1A1, and ACAN was not changed by the heat stimulus and their expression levels were lower than those in pellets cultured at 37 °C (Fig. 2a). There were also no significant differences in these mRNA expression levels between the 32 °C group and the 32 °C + Heat group on day 7, although COL2A1 expression in the 37 °C group was significantly higher than that in the 32 °C group and the 32 °C + Heat group (Fig. 2b).

#### Collagen and proteoglycan accumulation

**Fig. 3:**
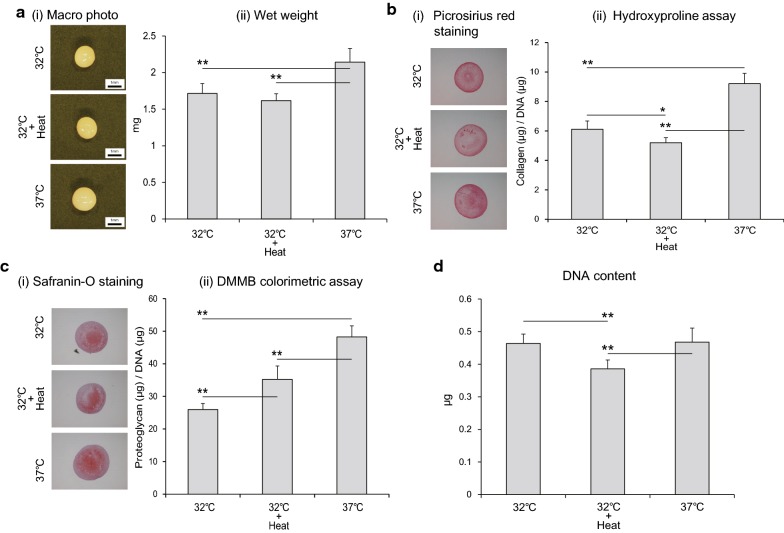
Collagen and proteoglycan accumulation. **a** Macro images and wet weight. Synthesized pellets on day 21 were subjected to macro photography (i) and wet weight measurement (ii). **b** Collagen accumulation. Collagen synthesis in pellets on day 21 was evaluated by picrosirius red staining (i) and hydroxyproline assay (ii). The values of collagen were normalized to DNA content. **c** Proteoglycan accumulation. Proteoglycan synthesis in pellets on day 21 was evaluated by safranin-O staining (i) and DMMB colorimetric assay (ii). The values of proteoglycan were normalized to DNA content. **d** DNA content. DNA content of pellets on day 21 was evaluated using the Quanti-iT™ PicoGreen assay. Values represent the means and standard deviations. * and ** indicates P < 0.05 and P < 0.01, respectively

The wet weight of the 37 °C group was significantly greater than that of the 32 °C group and the 32 °C + Heat group; however, there was no significant difference between the wet weight of the 32 °C group and the 32 °C + Heat group (Fig. 3a). The intensity of picrosirius red staining in the 32 °C + Heat group appeared to be low compared with that in other groups (Fig. 3b-i). In addition, the 32 °C + Heat group showed significantly lower hydroxyproline content per cell compared to the 32 °C group and the 37 °C group (Fig. 3b-ii). The intensity of safranin-O staining in the 37 °C group appeared to be higher than that in the other groups (Fig. 3c-i). The DMMB colorimetric assay showed that the proteoglycan content per cell in the 37 °C group was highest among the groups and that in the 32 °C + Heat group was significantly higher than that in the 32 °C group (Fig. 3c-ii). The DNA content in the 32 °C + Heat group was significantly lower than that in the other groups (Fig. 3d).

### Discussion

In this study, we investigated the effect of periodic mild heat stimuli on ECM synthesis of AC in pellet cultured human chondrocytes. We originally hypothesized that periodic mild heat stimulation enhances ECM synthesis; however, our results showed that in our study conditions, it diminished the accumulation of collagen, which is a major ECM of AC.

AC consists of three major components: type II collagen, aggrecan, and the AC specific cell type, chondrocytes [18]. We analyzed the immediate and accumulative effects of heat stimuli on the mRNA expression of COL2A1 and ACAN as well as COL1A1, which is a fibrocartilage marker. Heat stimulus of 41 °C for 20 min, which is considered a mild heat stimulus [19], did not affect the expression of these mRNAs in pellets cultured at 32 °C, mimicking the in vivo situation, though the mRNA expression of HSPA1A was upregulated. Compared with 37 °C, the 32 °C + Heat group as well as the 32 °C group showed lower mRNA expression of COL2A1, ACAN, and COL1A1, consistent with our previous reports [11]. These results indicate that there are few effects of periodic mild heat stimuli on the expression of these ECM related mRNAs in this experimental condition. We performed an additional experiment to confirm the effects of different temperature stimulus, and found that there were no positive effects of heat stimulus at 41–45 °C for 20 min on the expression of ECM-related mRNAs in pellet-cultured chondrocytes (see Additional file 2).

Biochemical assessment of the collagen and proteoglycan content per cell showed that collagen accumulation per cell was diminished in the 32 °C + Heat group. This would indicate that collagen synthesis was inhibited at the translational or post-translational level, but not at the transcription level, because there was no significant difference in the mRNA expression of COL2A1 and ACAN between the 32 °C group and the 32 °C + Heat group. It is reported that the triple-helix conformation of collagen cannot be formed at around 40 °C [20]. In addition, the DNA content in the 32 °C + Heat group was significantly lower than that in the other groups, indicating cell loss in the 32 °C + Heat group. We previously reported cell loss in the pellet culture system with continuous heat stimulation at 41 °C [10, 11], whereas little cell loss was observed in a monolayer culture system at the same temperature [21]. In our previous study, we mentioned that prolonged exposure to heat stimulation may have a negative effect, and that periodic heat stimulation may have a positive effect on ECM formation. However, the present study indicates that mild heat stimuli might diminish collagen synthesis and cause cell loss even if applied in a periodic manner. Further studies are necessary to elucidate the effect of heat stimuli on ECM synthesis using several microenvironmental conditions as well as implanted chondrocytes in vivo before applying heat stimuli for a clinical trial of ACI.

### Conclusion

Here we investigated the effects of periodic mild heat stimuli on ECM synthesis of adult human chondrocytes in a 3-D pellet culture system. Our results indicated that periodic mild heat stimuli could diminish ECM synthesis due to inhibition of the collagen production and loss of cells. Although application of a heat stimulus to enhance chondrocyte ECM synthesis would have potential as a method for ACI treatment, there are also associated risks as we indicate here. Further research is warranted for its practical application.

## Limitations

We identified four limitations in this study. First, our results were derived by assessing the chondrocytes obtained from one patient only. Therefore, our data could not be simply generalized, and needs confirmation using chondrocytes from a variety of backgrounds including age and disease. Second, we could not clarify the effects of the duration of heat stimulus. There is a possibility that shorter or longer heat stimulation might have a positive effect on ECM synthesis. Third, we utilized the pellet culture system for mimicking the in vivo condition. We considered that this system was suitable for this study because it is simple and provides reproducibility as well as simulation of regenerative or developmental processes of articular cartilage. However, this system could not completely simulate the in vivo environment. We have reported that different culture systems such as monolayer and pellet culture systems show different responses to culture temperature [22]. Similarly, chondrocytes in the pellet culture system might respond differently from chondrocytes in vivo. We focused on the effects of periodic heat stimuli during AC regeneration such as in ACI and utilized the pellet culture system to simplify but retain the 3-D culture condition in this study. Fourth, we did not measure the actual temperature within the pellets although we measured it in the culture medium. We consider that this does not affect our results because the synthesized pellets were small (minimum diameter of 1.4–1.7 mm on day 21) and because the thermal conductivity of water is high. Therefore, we consider that the thermal conditions on and in the pellet would show a similar transition with the culture medium as described in Fig. 1.

## Additional files

**Additional file 1.** Primer sequences for qRT-PCR.

**Additional file 2.** Effects of heat stimulation between 41 °C to 45 °C on the mRNA expression of HSPA1A, COL2A1, and ACAN. To confirm the effects of higher heat stimulation, we performed an additional experiment applying heat stimulus between 41 °C to 45 °C. The pre-cultured pellets were subjected to heat stimulation at 41 °C, 43 °C, or 45 °C for 20 min in a water bath. A shows the temperature transitions in a conical tube immersed in a water bath set at the specific temperature. After the heat stimulus, the mRNA expression of COL2A1, ACAN, and HSPA1A was analyzed at 3, 6, and 24 hours after the stimulus (B, C, and D). The pellets cultured at 32°C were used as controls (n = 3 pellets/time point). The results showed that HSPA1A expression was apparently upregulated, indicating successful heat stimulus (B). However, the 41°C and 43°C groups showed no positive effects on COL2A1 and ACAN expression (C and D). Transient upregulation of COL2A1 and ACAN was observed at 6 hours after stimulation in the 45°C group (C and D), but these were downregulated at 24 h after the stimulus.

## Abbreviations

AC: articular cartilage
ACI: autologous chondrocyte implantation
ECM: extracellular matrix
3-D: three-dimensional
HSPA1A: heat shock 70 kD protein 1A
COL2A1: type II collagen A1
COL1A1: type I collagen A1
ACAN: aggrecan
qRT-PCR: quantitative real-time reverse-transcription polymerase chain reaction
RPL13a: ribosomal protein L 13a
YWHAZ: tyrosine 3-monooxygenase/tryptophan 5-monooxygenase activation protein
DMMB: 1,9-dimethylmethylene blue
