# Supplementary material for: Periodic mild heat stimuli diminish extracellular matrix synthesis in pellet cultured human chondrocytes
Source: BMC Res Notes. 2019 Jan 14;12:16. doi: 10.1186/s13104-019-4058-x (PMC6332905; doi:10.1186/s13104-019-4058-x)
Supplement: Supplementary file 2 — Additional file 2. Effects of heat stimulation between 41 °C to 45 °C on the mRNA expression of HSPA1A, COL2A1, and ACAN. To confirm the effects of higher heat stimulation, we performed an additional experiment applying heat stimulus between 41 °C to 45 °C. The pre-cultured pellets were subjected to heat stimulation at 41 °C, 43 °C, or 45 °C for 20 min in a water bath. A shows the temperature transitions in a conical tube immersed in a water bath set at the specific temperature. After the heat stimulus, the mRNA expression of COL2A1, ACAN, and HSPA1A was analyzed at 3, 6, and 24 hours after the stimulus (B, C, and D). The pellets cultured at 32°C were used as controls (n = 3 pellets/time point). The results showed that HSPA1A expression was apparently upregulated, indicating successful heat stimulus (B). However, the 41°C and 43°C groups showed no positive effects on COL2A1 and ACAN expression (C and D). Transient upregulation of COL2A1 and ACAN was observed at 6 hours after stimulation in the 45°C group (C and D), but these were downregulated at 24 h after the stimulus. [file 13104_2019_4058_MOESM2_ESM.docx]

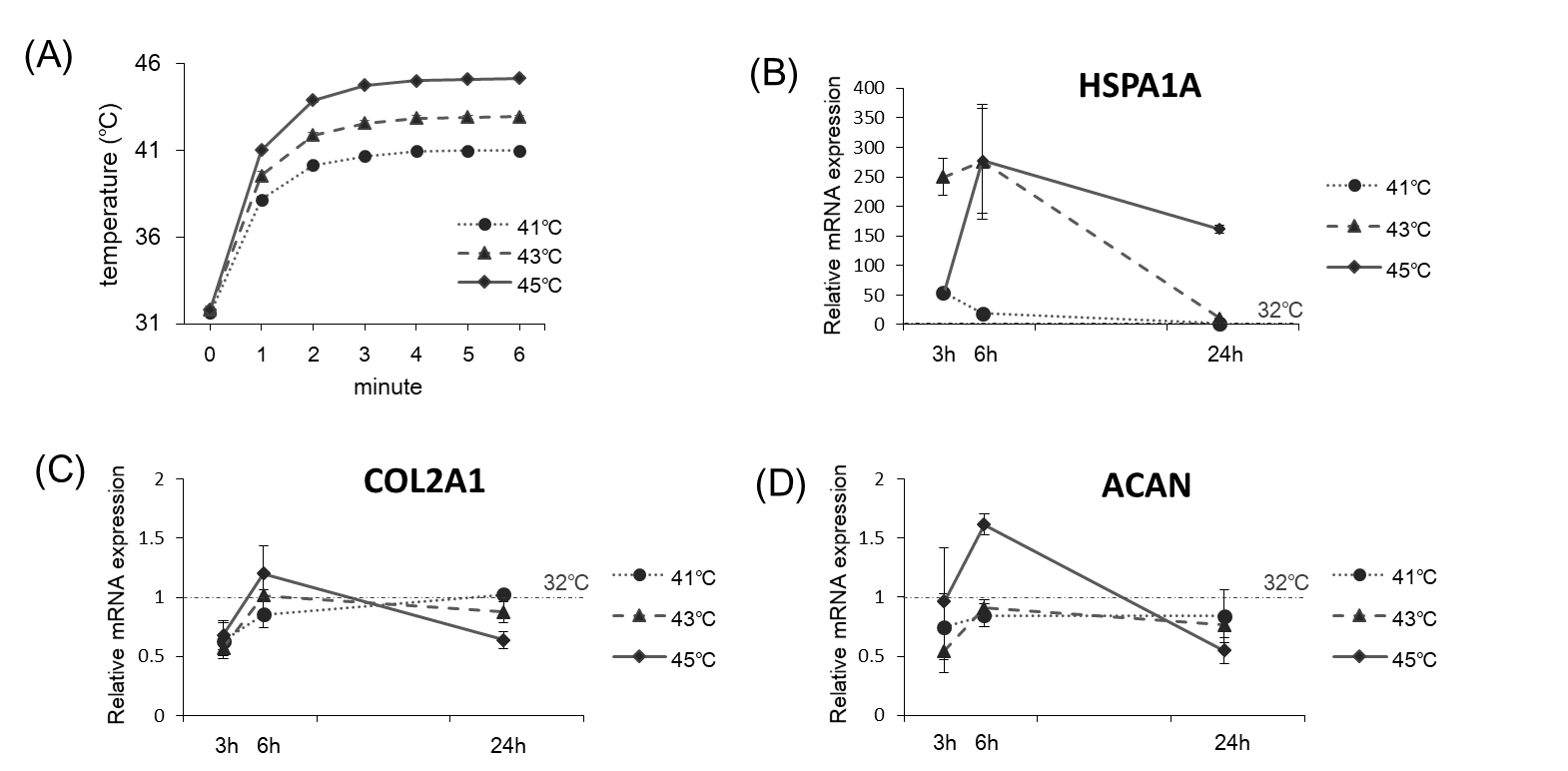


**Additional file 2.** Effects of heat stimulation between 41°C to 45°C on the mRNA expression of HSPA1A, COL2A1, and ACAN.

To confirm the effects of higher heat stimulation, we performed an additional experiment applying heat stimulus between 41°C to 45°C. The pre-cultured pellets were subjected to heat stimulation at 41°C, 43°C, or 45°C for 20 minutes in a water bath. Additional file 2A shows the temperature transitions in a conical tube immersed in a water bath set at the specific temperature. After the heat stimulus, the mRNA expression of COL2A1, ACAN, and HSPA1A was analyzed at 3, 6, and 24 hours after the stimulus (B, C, and D). The pellets cultured at 32°C were used as controls (n = 3 pellets/time point).

The results showed that HSPA1A expression was apparently upregulated, indicating successful heat stimulus (B). However, the 41°C and 43°C groups showed no positive effects on COL2A1 and ACAN expression (C and D). Transient upregulation of COL2A1 and ACAN was observed at 6 hours after stimulation in the 45°C group (C and D), but these were downregulated at 24 hours after the stimulus.
